# Risk of BIND and kernicterus in late preterm

**DOI:** 10.1186/1824-7288-40-S2-A11

**Published:** 2014-10-09

**Authors:** Carlo Dani

**Affiliations:** 1 Department of Neurosciences, Psychology, Drug Research and Children’s Health, University of Florence, Italy

## 

Severe hyperbilirubinemia can induce devastating and permanent neurodevelopmental handicaps in infants. The occurrence of hyperbilirubinemia is higher in late preterm than in term infants, ranging from 8 to 40% of the formers according to different definitions of hyperbilirubinemia. The mechanisms by which this occurs is not completely understood, however an increased bilirubin load on hepatocyte, as result of decreased erythrocyte survival and increased erythrocyte volume, increased enterohepatic circulation of bilirubin, decreased hepatic uptake of bilirubin from plasma, defective bilirubin conjugation, and diminished serum bilirubin binding capacity play a relevant role.

Hyperbilirubinemia in late preterm infants is not only more prevalent than in term neonates, but also it occurs later and is more severe and protracted. In fact, Maisels et al. (Pediatrics 2006;117:1169) demonstrated that at 72 hours of life the value of 50° percentile of trans-cutaneous bilirubin (TcB) is 9 mg/dl in 35-37 wks infants, while is <6 mg/dl in ≥40 wks infants. Moreover, they found that the decrease of TcB is slower in 35-37 wks infants than in ≥40 wks infants, since at 96 hours of life the value of 50° percentile of TcB is 9 mg/dl in 35-37 wks infants, while is <3 mg/dl in >40 wks infants.

Late preterm infants are at higher risk of bilirubin induced neurological dysfunction (BIND) and kernicterus than term infants. The mechanisms that potentially could explain the high susceptibility of central nervous system to bilirubin-induced damage in late preterm neonates have not been well defined. However, some of the factors that can potentially contribute are the diminished serum bilirubin binding capacity due to the lower serum albumin levels, an enhanced permeability of the blood–brain barrier to unconjugated bilirubin influx, and an immaturity of neuronal protective mechanisms. This is probably the reason why late preterm neonates are at an increased risk to develop acute bilirubin encephalopathy and or kernicterus as demonstrated in the USA pilot kernicterus registry (Semin Perinatol 2006;30:89) in which this category of infants is over-represented compared to term neonates.

Thus, clinicians need to be more concerned and conscientious to identify late preterm’s risk for severe hyperbilirubinemia in view of their increased susceptibility to BIND. This can allow of planning a prevention program including nursing and parental education, screening for jaundice in the nursery, the provision of lactation support, timely post discharge follow-up, and appropriate treatment when clinically indicated.

